# Distinct tumor immune microenvironments in primary and metastatic lesions in gastric cancer patients

**DOI:** 10.1038/s41598-020-71340-z

**Published:** 2020-08-31

**Authors:** Seung-Myoung Son, Chang Gok Woo, Dae Hoon Kim, Hyo Yung Yun, Hongsik Kim, Hee Kyung Kim, Yaewon Yang, Jihyun Kwon, Minsuk Kwon, Tae-Yong Kim, Hyung-Don Kim, June-Young Koh, Su-Hyung Park, Eui-Cheol Shin, Hye Sook Han

**Affiliations:** 1 Department of Pathology, Chungbuk National University Hospital, Cheongju, Republic of Korea; 2 Department of Pathology, Chungbuk National University College of Medicine, Cheongju, Republic of Korea; 3 Department of Surgery, Chungbuk National University Hospital, Cheongju, Republic of Korea; 4 Department of Surgery, Chungbuk National University College of Medicine, Cheongju, Republic of Korea; 5 Department of Internal Medicine, Chungbuk National University Hospital, Cheongju, Republic of Korea; 6 Department of Internal Medicine, Chungbuk National University College of Medicine, Chungdae-ro 1, Seowon-gu, Cheongju, 28644 Republic of Korea; 7 Division of Hematology-Oncology, Department of Internal Medicine, Samsung Medical Center, Sungkyunkwan University School of Medicine, Seoul, Republic of Korea; 8 Department of Internal Medicine, Seoul National University Hospital, Seoul National University College of Medicine, Seoul, Republic of Korea; 9 Department of Internal Medicine, Asan Medical Center, University of Ulsan College of Medicine, Seoul, Republic of Korea; 10 Laboratory of Immunology and Infectious Diseases, Graduate School of Medical Science and Engineering, Korea Advanced Institute of Science and Technology, 291 Daehak-ro, Yuseong-gu, Daejeon, 34141 Republic of Korea

**Keywords:** Cancer, Immunology, Oncology

## Abstract

This study compared the tumor immune microenvironments (TIMEs) of primary gastric cancer (PGC) and paired metastatic gastric cancer (MGC). CD4^+^ and CD8^+^ T-cell density and PD-L1 expression were evaluated by multiplex immunohistochemistry, DNA mismatch repair (MMR) by immunohistochemistry, and immune-related genes by RNA sequencing. Twenty-three patients who underwent surgical treatment for PGC and MGC were enrolled in this study. CD8^+^ T-cell, PD-L1^+^ cell, and PD-L1^+^CK^+^ cell densities were significantly lower in MGC than PGC. PD-L1 positivity using a combined positive score (≥ 1%) and deficient MMR were observed in 52.2% and 8.7% of PGC samples, respectively, whereas both occurred in only 4.3% of MGC samples. The most frequent TIME types were inflamed (34.8%) and adaptive immune resistance (34.8%) in PGC, and immune desert (65.2%) and immunological ignorance (73.9%) in MGC. In transcriptome analysis, the expression of the T-cell inflamed gene set and co-stimulatory gene module was down-regulated in MGC compared to PGC. The total CD8^+^ T-cell density was an independent prognostic marker in both PGC and MGC (univariate *P* = 0.002, multivariate *P* = 0.006). Our result suggest that the TIME of metastatic tumors was less immunologically active compared to that of primary tumors in gastric cancer patients.

## Introduction

Palliative systemic therapy is a primary treatment option in patients with metastatic gastric cancer (MGC) to alleviate disease-related symptoms and improve survival^1,2^. However, the prognosis of MGC is still very poor, with a survival of < 2 years^1,2^. Recently, immune checkpoint inhibitors (ICIs), such as anti-programmed cell death 1 (PD-1) and anti-programmed death-ligand 1 (PD-L1), have revolutionized the systemic treatment paradigm in many advanced solid tumors^3^. Anti-PD-1 antibodies, such as nivolumab and pembrolizumab, have significant benefits and offer new treatment options in patients with previously treated MGC^4–6^. However, the overall response rate for anti-PD-1 antibodies in patients with previously treated MGC is low (5–25%), and most patients do not respond to these ICIs^4–6^. Although no definite predictive biomarkers of anti-PD-1 or PD-L1 antibodies are available, PD-L1 expression, DNA mismatch repair (MMR), and Epstein-Barr virus (EBV) status have been proposed as predictive biomarkers of the ICI response in MGC patients^7^. Moreover, high tumor-infiltrating lymphocyte (TIL) levels have been reported as a favorable prognostic biomarker and significant predictor of immunotherapy^8–10^.

Anti-PD-1 treatment is a systemic therapy for metastatic disease in patients with MGC who have previously received cytotoxic or molecularly targeted agents. A comprehensive evaluation of immune-related biomarkers in metastatic tumors is needed before starting anti-PD-1 treatment. However, re-biopsy of a metastatic lesion is not always feasible in clinical practice and could result in unpleasant complications due to invasive procedures. Therefore, the assessment of immune-related biomarkers for MGC was based on the tumor immune microenvironment (TIME) of primary gastric cancer (PGC) specimens obtained during curative resected gastrectomy before recurrence, or PGC specimens acquired at the time of initial diagnosis before palliative systemic therapy. Since the TIME of metastatic tumors may differ from that of primary tumors due to intratumoral (IT) or inter-tumoral molecular heterogeneity, immune escape during metastasis or disease progression, and systemic effects of previous therapy, PGC specimens may not accurately reflect the TIME of MGC. However, no previous report has evaluated immune-related biomarkers in MGC specimens.

In this study, we compared the TIMEs of primary and paired metastatic gastric cancer, including T-cell density, PD-L1 expression, MMR status, EBV positivity, and immune-related gene expression. We also evaluated the prognostic impact of tumor-infiltrating T cells in primary and metastatic tumors in gastric cancer patients.

## Results

### Clinicopathological characteristics

We screened a pathological database for 120 patients with pathologically confirmed gastric adenocarcinoma in primary lesions and corresponding metastatic lesions between January 2009 and May 2019. Of these patients, 23 patients who underwent surgical treatment for PGC and MGC were enrolled in this analysis (Supplementary Fig. 1). The clinicopathological characteristics of the patients are presented in Table 1. Surgically resected metastatic sites were the liver (n = 6, 26.1%), abdominal wall or skin (n = 5, 21.7%), ovary (n = 4, 17.4%), and other various metastatic sites, including gallbladder, lung, distant lymph node, bone, peritoneum, or appendix (n = 8, 34.8%). Of the 23 patients, only 7 patients (30.4%) had stage IV disease at initial diagnosis of gastric cancer and received simultaneous resection including gastrectomy and MGC resection. Sixteen patients (69.6%) had metachronous metastasis and receive staged resection including gastrectomy and MGC resection at 6 months or more after gastrectomy. The median disease-free survival of these 16 patients was 21.0 months and the duration between gastrectomy and MGC resection was 23.1 months. Further, 5 of these 16 patients received systemic chemotherapy before MGC resection.

**Table 1:** Clinicopathological characteristics.

	No	%
**Age at metastatic disease diagnosis, years**		
Median	60 (range 39–78)	
**Sex**		
Male	15	65.2
Female	8	34.8
**Timing of metastatic disease**		
Synchronous	7	30.4
Metachronous	16	69.6
**Extent of metastatic disease**		
Number of metastatic sites		
1	13	56.5
2–3	10	43.5
Presence of peritoneal metastasis		
No	17	73.9
Yes	6	26.1
**Palliative systemic therapy**		
No	4	17.4
1st or 2nd line	12	52.2
3rd or 4th line	7	17.4
**PGC**		
Primary tumor location		
Gastroesophageal junction/fundus	1	4.4
Body	7	30.4
Antrum	15	65.2
Histological grade		
Good	11	47.8
Poor^a^	12	52.2
**MGC**		
Surgically resected metastatic site		
Liver	6	26.1
Abdominal wall or skin mass	5	21.7
Ovary	4	17.4
Others	8	34.8
Duration between gastrectomy and MGC resection		
Simultaneous resection	7	30.4
Staged resection (≥ 6 months)	16	69.6
Systemic therapy within 1 year of MGC resection		
Yes	5	21.7
No	18	78.3

*PGC* primary gastric cancer, *MGC* metastatic gastric cancer.

^a^Poor histological grading defined as poorly differentiated tubular adenocarcinoma or poorly cohesive carcinoma according to the WHO classification.

A comparison of PD-L1 positivity, MMR status, and EBV positivity between PGC and MGC is shown in Table 2. Using a 1% cut-off value for combined positive score (CPS), PD-L1 positivity was observed in 52.2% (12/23) of PGC cases and 4.3% (1/23) of MGC cases. Deficient MMR (dMMR) was detected more frequently in PGC (8.7%, 2/23) than paired MGC (4.3%, 1/23). One of the patients had PGC with dMMR and a synchronous skin metastatic tumor with proficient MMR (pMMR) (Supplementary Fig. 2). EBV was not detected in 46 PGC and MGC specimens from 23 patients.

**Table 2:** Concordance of PD-L1 positivity, MMR status, and EBV positivity between PGC and MGC.

	PGC		Total
MGC	Negative	Positive	
**PD-L1 positivity (CPS** **≥** **1%)**			
Negative	11	11	22 (95.7)
Positive	0	1	1 (4.3)
Total	11 (47.8)	12 (52.2)	23 (100.0)
**MMR status**			
pMMR	21	1	22 (95.7)
dMMR	0	1	1 (4.3)
Total	21 (91.3)	2 (8.7)	23 (100.0)
**EBV positivity**			
Negative	23	0	23 (100.0)
Positive	0	0	0
Total	23 (100.0)	0	23 (100.0)

Data are given as number of patients or n (%).

*PD-L1* programmed death-ligand 1, *MMR* DNA mismatch repair, *EBV* Epstein–Barr virus, *PGC* primary gastric cancer, *MGC* metastatic gastric cancer, *CPS* combined positive score, *dMMR* deficient MMR, *pMMR* proficient MMR.

### Comparison of T-cell density and PD-L1 expression between PGC and MGC

**Figure 1:**
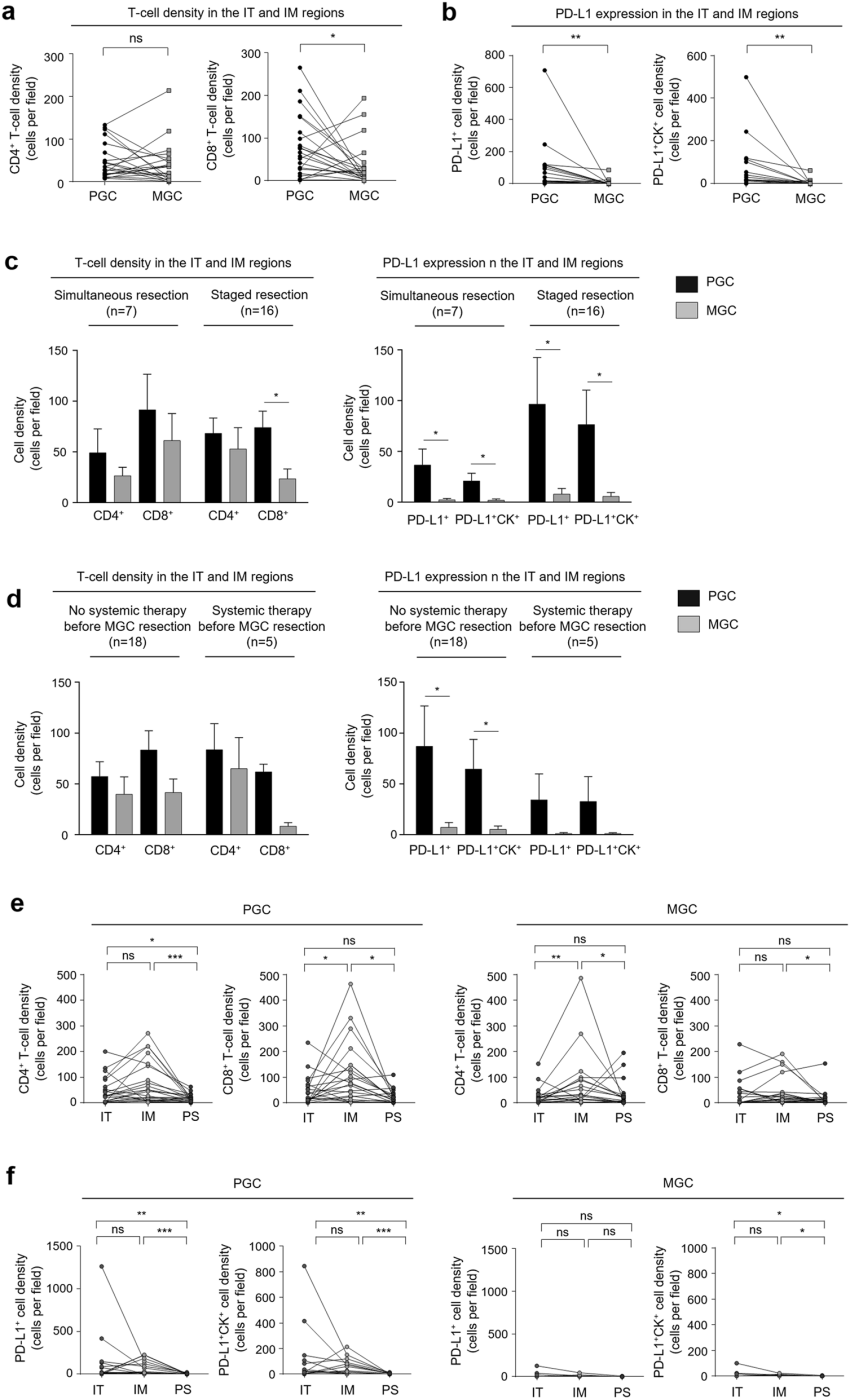
Tumor-infiltrating T-cell density and PD-L1 expression in PGC and MGC. (**a)** Comparison of CD4^+^ and CD8^+^ T-cell density in the IT and IM regions. (**b)** Comparison of PD-L1^+^ and PD-L1^+^CK^+^ cell density in the IT and IM regions. **(c)** T-cell density and PD-L1 expression in the IT and IM regions analyzed according to the duration between gastrectomy and MGC resection and (**d)** whether patients received systemic therapy before MGC resection. (**e)** CD4^+^ and CD8^+^ T-cell density and (**f)** PD-L1^+^ and PD-L1^+^CK^+^ cell density according to tumor region. *PGC* primary gastric cancer, *MGC* metastatic gastric cancer, *IT* intratumoral, *IM* invasive margin, *PD-L1* programmed death-ligand 1, *CK* cytokeratin. Statistical analyses were performed using the Wilcoxon signed rank test (*ns* not significant; *P < 0.05; **P < 0.01; ***P < 0.001).

T-cell density and PD-L1 expression in PGC and MGC are shown in Supplementary Table 1. CD4^+^ T-cell density in the IT and invasive margin (IM) regions was lower in MGC (44.5 ± 71.0 cells/field) than in PGC (62.2 ± 60.2 cells/field), but the difference was not significant (*P* = 0.200; Fig. 1a). CD8^+^ T-cell density in the IT and IM regions was significantly lower in MGC (35.5 ± 52.8 cells/field) than in PGC (79.6 ± 73.1 cells/field; *P* = 0.046; Fig. 1a), as was PD-L1^+^ cell density (6.2 ± 18.6 cells/field vs. 77.5 ± 154.0 cells/field; *P* = 0.010; Fig. 1b). PD-L1^+^CK^+^ cell density in the IT and IM regions was also significantly lower in MGC (4.5 ± 13.3 cells/field) than in PGC (58.8 ± 114.7 cells/field; *P* = 0.001; Fig. 1b). The lower T-cell density and PD-L1 expression in the IT and IM regions in MGC was observed regardless of the duration between gastrectomy and MGC resection (Fig. 1c) or systemic therapy initiation before MGC resection (Fig. 1d). In the three regions including peritumoral stromal (PS), T-cell density and PD-L1 expression exhibited similar trends as T-cell density and PD-L1 expression in the IT and IM regions (Supplementary Table 1).

A regional comparison for each tumor showed that CD4^+^ and CD8^+^ T-cell density were higher in the IM regions than in the IT and PS regions (*P* = 0.016 for CD8^+^ T-cell density in PGC, and *P* = 0.008 for CD4^+^ T-cell density in MGC; Fig. 1e). PD-L1 expression tended to be higher in the IT regions than the IM and PS regions, though the difference was not significant in PGC and MGC (Fig. 1f).

### Comparison of TIME types between PGC and MGC

**Figure 2:**
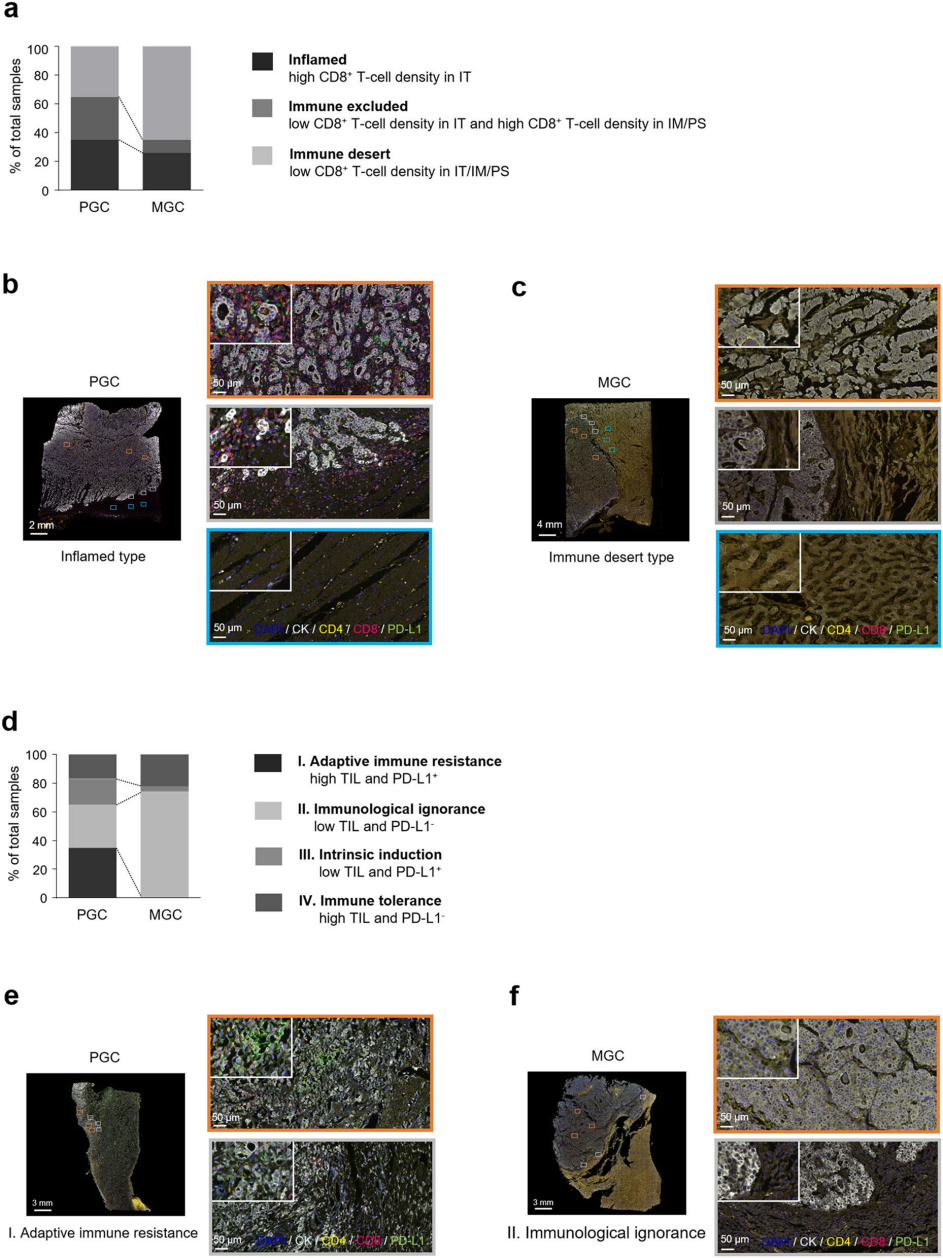
Comparison of tumor immune microenvironment types between PGC and MGC. (**a**) Comparison of types based on CD8^+^ T-cell density and the distribution between PGC and MGC. (**b**) Representative multiplex IHC images of a PGC specimen with inflamed type (high CD8^+^ T-cell density in the IT region). (**c**) Representative multiplex IHC images of paired MGC specimens with immune desert type (low CD8^+^ T-cell density in the IT, IM, and PS regions). (**d**) Comparison of types based on T-cell density and PD-L1 expression in the IT and IM regions between PGC and MGC. (**e**) Representative multiplex IHC images of PGC specimens with adaptive immune resistance type (high CD4^+^ + CD8^+^ T-cell density and PD-L1-positive). (**f**) Representative multiplex IHC images of paired MGC specimens with immunological ignorance type (low CD4^+^ + CD8^+^ T-cell density and PD-L1-negative). *PGC* primary gastric cancer, *MGC* metastatic gastric cancer, *IHC* immunohistochemistry, *IT* intratumoral, *IM* invasive margin, *PS* peritumoral stroma, *PD-L1* programmed death-ligand 1, *CK* cytokeratin.

PGC and MGC specimens were categorized into three types based on CD8^+^ T-cell density and distribution within the tumor microenvironment (Supplementary Table 2, Fig. 2a)^11^. Inflamed type was observed in 34.8% (Fig. 2b) and 26.1%, immune excluded type in 30.4% and 8.7%, and immune desert type in 34.8% and 65.2% (Fig. 2c) of PGC and paired MGC specimens, respectively.

PGC and MGC specimens were also classified into four types based on the TIL density and PD-L1 expression: type I–adaptive immune resistance, type II–immunological ignorance, type III–intrinsic induction, and type IV–immune tolerance (Supplementary Table 2, Fig. 2d)^12^. PGC specimens had a higher proportion (34.8%) of type I than other subtypes (Fig. 2e). In the paired MGC specimens, we found no type I cases and a higher proportion of type II (73.9%) compared to the other subtypes (Fig. 2f).

### Comparison of immune-related gene expression

**Figure 3:**
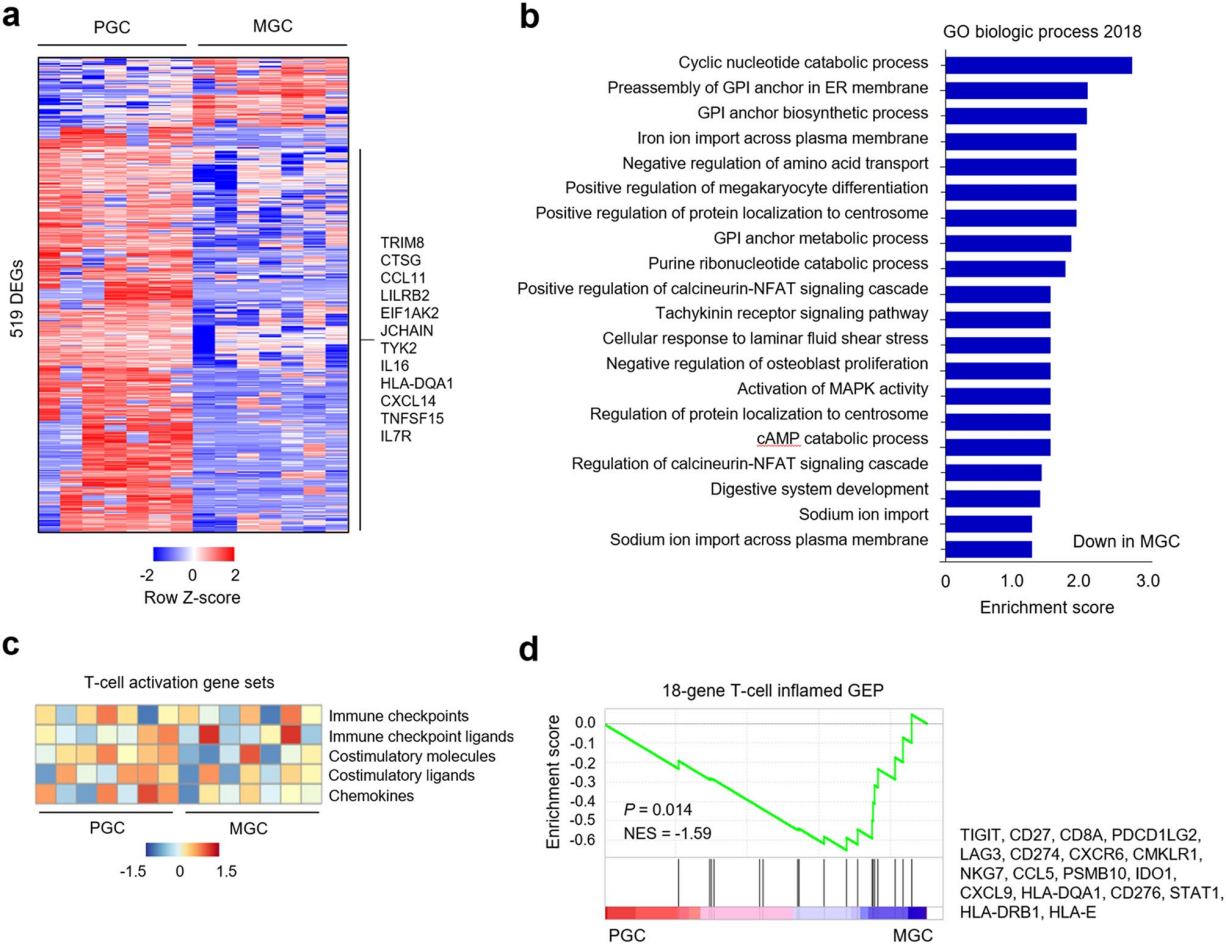
Immune-related gene expression profiles. (**a**) Heat map of 519 differentially expressed genes (DEGs; *P* < 0.05 and fold change > 2). (**b**) Gene ontology analysis of the DEGs. (**c**) Gene Set Validation Analysis to assess the enrichment of gene sets related to immune checkpoints, immune checkpoint ligands, co-stimulatory molecules, co-stimulatory ligands, and chemokines. (**d**) Gene Set Enrichment Analysis to assess the enrichment of 18-gene T-cell inflamed gene expression profiles. *PGC* primary gastric cancer, *MGC* metastatic gastric cancer, *NSE* normalized enrichment score, *GEP* gene expression profile.

RNA sequencing revealed that a total of 76 genes were significantly up-regulated and 443 genes significantly down-regulated in MGC compared to PGC (Fig. 3a). The immune-related genes that were down-regulated in MGC compared to PGC included several genes associated with the innate immune response (i.e., *TRIM8*, *CTSG*, *CCL11*, *LILRB2*, and *EIF1AK2*), immunoglobulin structure (i.e., *JCHAIN*), and T-cell activation (i.e., *TYK2*, *IL16*, *HLA-DQA1*, *CXCL14*, *TNFSF15*, and *IL7R*). Gene Ontology term analysis revealed that T-cell activation-related genes involved in calcineurin-nuclear factor of activated T cells (NFAT) and mitogen-activated protein kinase (MAPK) signaling cascades were down-regulated in MGC compared to PGC (Fig. 3b). Gene Set Validation Analysis (GSVA) for T-cell activation-related gene sets showed that the co-stimulatory molecule gene module decreased in MGC compared to PGC (Fig. 3c). Next, we performed Gene Set Enrichment Analysis (GSEA) using 18-gene T-cell inflamed gene expression profile (GEP) to predict responses to PD-1 blockade^13,14^ and found that the T-cell inflamed GEP decreased in MGC compared to PGC (Fig. 3d).

### Prognostic impact on overall survival

**Figure 4:**
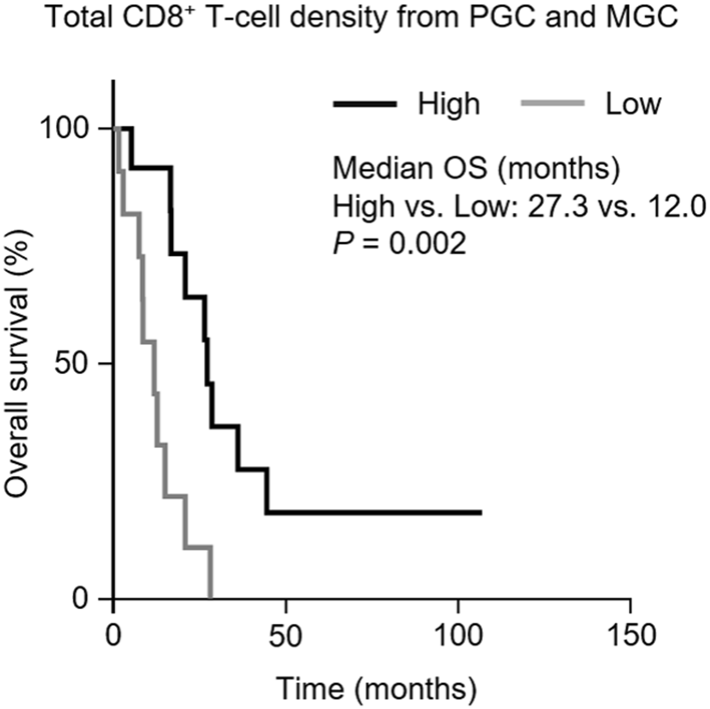
Overall survival according to total CD8^+^ T-cell density in PGC and MGC. *PGC* primary gastric cancer, *MGC* metastatic gastric cancer, *OS* overall survival. Statistical analysis was performed using the Kaplan–Meier method.

The median overall survival (OS) for all patients was 16.9 months (95% CI 10.5–23.2 months). Univariable and multivariable analyses for OS according to clinicopathological characteristics and tumor-infiltrating T-cell infiltration are summarized in Table 3. We grouped the patients into low and high T-cell density by the median number of CD8^+^ T cells per field (50.9 cells/field) and the number of CD4^+^ and CD8^+^ T cells per field (111.4 cells/field) in the IT and IM regions of PGC and MGC. Univariate analysis revealed that an absence of peritoneal metastasis (*P* = 0.073), high CD4^+^ + CD8^+^ T-cell density in PGC (*P* = 0.092), and high CD8^+^ T-cell density in PGC (*P* = 0.068) exhibited a trend towards improved OS, though the differences were not significant. We calculated the total CD8^+^ T-cell density by summing CD8^+^ T-cell densities from PGC and MGC tumors for each patient. The median OS was significantly longer in patients with high CD8^+^ T-cell density than patients with low CD8^+^ TIL density (27.3 vs. 12.0 months, respectively, *P* = 0.002; Fig. 4). Multivariable analysis included variables that tended to associate with survival (*P* < 0.1), and revealed that only the total CD8^+^ T-cell density was an independent prognostic factor for both PGC and MGC tumors (hazard ratio [HR] 0.188, 95% confidence interval [CI] 0.057–0.624, *P* = 0.006; Table 3).

**Table 3:** Univariable and multivariable analyses of overall survival.

Characteristic	No. of patients	Univariable analysis				Multivariable analysis		
		Median OS, months	HR	95% CI	*P *value	HR	95% CI	*P* value
**Age**					0.321			
< 60 years	11	16.9	1.618	0.626–4.183				
≥ 60 years	12	20.9						
**Histological grade**					0.823			
Good	11	16.7	1.114	0.433–2.869				
Poor	12	20.9						
**Timing of metastatic disease**					0.984			
Synchronous	7	20.9	0.990	0.358–2.734				
Metachronous	16	12.8						
**No. of metastatic sites**					0.552			
1	13	20.9	0.755	0.299–1.908				
2–3	10	15.0						
**Peritoneal metastasis**					0.073			0.151
No	17	20.9	0.322	0.093–1.112				
Yes	6	8.6				2.542	0.711–9.092	
**Palliative systemic treatment**					0.405			
No	4	8.6						
1st and 2nd line	12	15.0	0.997	0.239–4.155				
3rd and 4th line	7	20.9	0.512	0.120–2.173				
**CD4**^**+**^ **+** **CD8**^**+**^** T-cell density in PGC**					0.092			0.444
High	12	26.5	0.448	0.177–1.139		0.421	0.046–3.867	
Low	11	12.8						
**CD4**^**+**^ **+** **CD8**^**+**^** T-cell density in MGC**					0.724			
High	5	27.3	0.836	0.309–2.262				
Low	18	16.7						
**CD4**^**+**^ **+** **CD8**^**+**^** T-cell density in PGC and MGC**					0.312			
High	12	26.5	0.597	0.220–1.622				
Low	11	15.0						
**CD8**^**+**^** T-cell density in PGC**					0.068			0.578
High	13	26.5	0.406	0.154–1.068		1.965	0.184–21.290	
Low	10	8.6						
**CD8**^**+**^** T-cell density in MGC**					0.628			
High	5	27.3	0.784	0.294–2.096				
Low	18	16.7						
**CD8**^**+**^** T-cell density in PGC and MGC**					0.002			0.006
High	12	27.3	0.170	0.055–0.526		0.188	0.057–0.624	
Low	11	12.0						

*OS* overall survival, *HR* hazard ratio, *CI* confidence interval, *PGC* primary gastric cancer, *MGC* metastatic gastric cancer.

## Discussion

To the best of our knowledge, this study is the first to evaluate TIME differences between primary and pair-matched metastatic tumors in gastric cancer. We found that, in gastric cancer patients, immune-related biomarkers, such as T-cell density, dMMR status, PD-L1 expression, and the T-cell activation-related gene signature, were significantly lower in metastatic tumors than in primary tumors. Furthermore, a high CD8^+^ T-cell density of primary and metastatic tumors was associated with improved survival in MGC patients.

Many previous studies have reported discrepancies in T-cell infiltration and PD-L1 expression between primary and metastatic lesions in other solid tumors^15–20^. In our study, PGC has higher T-cell density, particularly CD8^+^ T cells and PD-L1 expression, than MGC. These differences between primary and metastatic tumors can be due to many factors, including the immune escape mechanisms of the metastatic tumor itself, time interval between primary and metastatic cancer tissue acquisition, and previous systemic therapy. However, according to our subgroup analysis, the T-cell density and PD-L1 expression were lower in MGC than PGC, regardless of the timing of MGC resection or systemic therapy before MGC resection. Therefore, the difference in TIME may be attributed to the immune escape mechanism of metastatic tumors and the selection pressure during disease progression or previous therapy.

In recent years, several classifications have been used to evaluate immunological status and predict anti-tumor responses to ICIs^11,12^. In our study, PGC specimens had almost equal proportions of inflamed (34.8%), excluded (30.4%), and immune desert (34.8%) types, whereas two-thirds (65.2%) of the MGC specimens were composed of the immune desert type. Moreover, the PGC specimens had a higher adaptive immune resistance type (34.8%) than other subtypes, and MGC specimens were almost all immunological ignorance type (73.9%). Two mechanisms underlying PD-L1 upregulation have been proposed: an intrinsic oncogenic activation mechanism, and an extrinsic adaptive immune-induced PD-L1 upregulation secondary to interferon-γ secreted by TILs^12,21^. As most gastric cancers are associated with inflammation^22^, PD-L1 expression in PGC could be upregulated by an extrinsic adaptive immune response. This supposition is supported by previous studies showing that PD-L1 upregulation may be feedback from active immune reactions rather than a result of neoantigen expression in gastric and colorectal cancer^23–25^. However, our results demonstrate that the T-cell density was lower in MGC than PGC, and PD-L1 expression was nearly absent in MGC. Therefore, in MGC, lymphocyte infiltration induced by inflammation, as observed in PGC, is very rare, and little PD-L1 expression occurs from extrinsic adaptive immune reactions, resulting in immune desert and immune ignorant types.

An improved understanding of the TIME in MGC and PGC may be useful for designing immunotherapy strategies to improve treatment outcomes in MGC patients. Adaptive immune resistance type (high TIL density and PD-L1-positive) will most likely benefit from anti-PD-1 or PD-L1 treatment, whereas a single-agent anti-PD-1 or PD-L1 regimen would most likely not be successful in immunological ignorance type (low TIL density and PD-L1-negative)^12^. dMMR/high microsatellite instability or high T-cell inflamed GEP predict an objective and durable response to anti-PD-1 or PD-L1 antibody across multiple solid tumors^7,13,14,26,27^. In our study, MGC exhibited lower T-cell density, PD-L1 expression, dMMR, and T-cell inflamed GEP than PGC. Therefore, the low response rate of anti-PD-1 in the previous clinical trials of MGC patients, despite PD-L1-positive tumor in archival PGC tissues, may be due in part to these TIME differences between PGC and MGC. Furthermore, the T-cell density was lower in the IT region than the IM regions in both PGC and MGC, as indicated by the influence of immunosuppressive stroma, myeloid-derived suppressor cells, and angiogenesis, which prevent T-cell infiltration into the tumors and suppress T-cell activation. Therefore, combination strategies with immunotherapy other than anti-PD-1/anti-PD-L1 or chemotherapy and molecular targeted therapy may induce lymphocyte infiltration and be effective in MGC patients. Recent studies have reported that combining anti-PD-1 with other therapies, such as ipilimumab, chemotherapy, or ramucirumab has a higher response rate in MGC patients than anti-PD-1 alone^28–31^.

Many studies have reported that TILs are associated with a favorable outcome in numerous types of solid tumors^8–10^. In particular, high TILs, mainly CD8^+^ T cells, may be a potential prognostic biomarker in patients with gastric cancer^8,24,25^. Although it is difficult to compare the prognostic impact of CD8^+^ T-cell density between PGC and MGC because only five patients had MGC with high CD8^+^ T-cell density, the total CD8^+^ T-cell density in both PGC and MGC predicted the prognosis more accurately than the CD8^+^ T-cell density in PGC alone. Previous studies have reported that TIL density in distant metastatic lesions could be a prognostic biomarker^17,32^. Therefore, the TIME of metastatic tumors should be considered an important prognostic parameter for predicting prognosis in MGC patients.

The present study has some limitations. First, this study was a retrospective analysis with a limited number of paired PGC and MGC specimens. It is difficult to obtain tumor specimens from the metastatic site in MGC patients. Most previous studies comparing T-cell infiltration or PD-L1 expression between primary and metastatic tumors were performed in colorectal cancer or renal cell carcinoma^23,32^, in which metastasectomy is the standard treatment to improve survival, or in breast or non-small cell lung cancer, for which re-biopsy is recommended in clinical practice to inform the palliative systemic therapy regimen^15,17,19^. However, surgical resection or re-biopsy at metastatic lesions is rarely performed in routine practice in MGC patients. Our study only included surgically resected tumor tissues to perform an accurate and comprehensive comparison of the TIMEs in PGC and MGC. Therefore, our results are meaningful, even though the sample number is small. Second, we could not evaluate detailed immune cell subsets, differentiation, or exhaustion status of TILs. Therefore, additional studies detailing differences in the TIME between primary and metastatic tumors using flow cytometry analysis and fresh tissue specimens or high-fidelity techniques, such as mass cytometry and single-cell RNA sequencing, are required. Third, we could not evaluate the correlation between the distinct TIMEs in primary and metastatic lesions and tumor response to immunotherapy, which is the rationale for evaluating TIME. However, it has recently been shown that immune heterogeneity may contribute to the different tumor responses to anti-PD-1 treatment in multifocal hepatocellular carcinoma^33^. Therefore, further studies are needed in the future to determine the association between TIME heterogeneity and immunotherapy treatment outcomes.

In conclusion, we suggest that the TIME of metastatic tumors is less immunologically active than that of primary tumors in MGC patients. Therefore, an evaluation of immune-related biomarkers in metastatic tumors, as well as primary tumors, should be considered when assessing the TIME in MGC patients.

## Methods

### Patients and tumor samples

Cases were reviewed using a database of patients pathologically confirmed to have primary and metastatic gastric adenocarcinoma at Chungbuk National University Hospital between January 2009 and May 2019. We collected surgically resected formalin-fixed paraffin-embedded (FFPE) tissue samples to comprehensively and accurately assess the TIME around the tumor. Two experienced pathologists evaluated hematoxylin and eosin-stained slides from each FFPE block and selected the slide with the largest viable tumor cell area. Clinicopathological data, including age, sex, timing of metastatic disease, number of metastatic sites, presence of peritoneal metastasis, palliative systemic therapy, PGC location, and World Health Organization histological classification, were obtained from medical records and pathology reports. The study protocol was in line with ethical guidelines of the 1975 Declaration of Helsinki. This study was reviewed and approved by the Institutional Review Board (IRB) of Chungbuk National University Hospital (CBNUH 2018-10-007), and the IRB waived the requirement to obtain written informed consent from the patients.

### Multiplex immunohistochemical staining protocol

T-cell density and PD-L1 expression were evaluated by multiplex immunohistochemistry (IHC) using the OPAL multiplex IHC kit (Perkin-Elmer, Waltham, MA) with a panel consisting of T-cell markers (CD4 and CD8), an immune checkpoint molecule (PD-L1), tumor cell marker (cytokeratin [CK]), and 4′,6-diamidino-2-phenylindole (DAPI) for nuclear DNA. The FFPE tissue sections (4-μm-thick) were heated for at least 1 h in a dry oven at 60 °C and dewaxed using xylene. The sections were then stained using a Leica Bond Rx Automated Stainer (Leica Biosystems, Newcastle, UK). The slides were baked for 30 min and dewaxed with Leica Bond Dewax solution (#AR9222, Leica Biosystems). Antigen retrieval was performed using Bond Epitope Retrieval 2 pH 9.0 buffer (#AR9640, Leica Biosystems) for 30 min. The slides were incubated with primary antibodies for rabbit anti-CD4 (ab133616, Abcam, diluted 1:100), mouse anti-CD8 (MCA1817T, BIO-RAD, diluted 1:300), rabbit anti-PD-L1 (13684S, Cell Signaling Technologies, diluted 1:300), and mouse anti-CK (M3515, Dako, diluted 1:300) for 30 min, followed by detection with Polymer HRP Ms + Rb (ARH1001EA, Perkin-Elmer). CD4, CD8, and PD-L1-stained slides were incubated with Opal 480 TSA Plus (diluted 1:150), Opal 520 TSA Plus (diluted 1:150), and Opal 620 TSA Plus (diluted 1:150), respectively, for 10 min. Each slide was then treated with Bond Epitope Retrieval 1 buffer (#AR9961, Leica Biosystems) for 20 min to remove bound antibodies before the next step in the sequence. CK-stained slides were incubated with Opal TSA-DIG Reagent (FP1502, Perkin-Elmer, diluted 1:70) for 10 min and Opal 780 TSA Plus (diluted 1:25) for 60 min. Nuclei were subsequently stained with DAPI and the section coverslipped using HIGHDEF IHC fluoromount (ADI-950-260-0025, Enzo). The slides were imaged using a PerkinElmer Vectra Polaris Automated Quantitative Pathology Imaging System (Perkin-Elmer) and analyzed using inForm 2.4.4 software and TIBCO Spotfire (Perkin-Elmer).

### Multiplex IHC data analysis

All slides were scanned at 10× magnification to select regions of interest (ROIs) using Phenochart software (ver. 1.0.10, Perkin-Elmer). ROIs included IT (within the tumor parenchyma), IM (within the lesion and approximately 500 µm on each side of the tumor margin), and PS (within the peritumoral lesion but outside the IM) regions (Supplementary Fig. 3). Three non-overlapping fields (one microscopic field, 925 × 693 µm) were selected from each region. The total number of cells in each region was calculated by the sum of the number of immunoreactive cells in three selected fields per region. The results were expressed as the mean density (cells per field) of positively stained cells.

### MMR status determination and EBV in situ hybridization

Fully automated immunostaining was performed on 4-μm-thick FFPE sections using a BenchMark XT autostainer (Ventana Medical Systems, Tucson, AZ). IHC for MMR proteins was performed according to standard antibody protocols using the following antibodies: mouse anti-MLH1 (Ventana/Roche M1, pre-dilute antibody), mouse anti-MSH2 (Roche G219-1129, pre-dilute antibody), rabbit anti-MSH6 (Roche SP93, pre-dilute antibody), and mouse anti-PMS2 (Roche A16-4, pre-dilute antibody). Tumors lacking MLH1, MSH2, PMS2, or MSH6 expression were considered dMMR, whereas tumors that expressed MLH1, MSH2, PMS2, or MSH6 were considered pMMR.

EBV status was determined by in situ hybridization with EBV-encoded small RNA (EBER) probes (INFORM EBER, Roche, ready-to-use) according to product protocols. Strong EBER signals were interpreted as EBV-positive.

### Classification of the TIME

Tumors were classified into three types based on CD8^+^ T-cell density in the IT, IM, and PS regions to characterize the pattern of immune activity^11^: inflamed (high CD8^+^ T-cell density in the IT region), immune-excluded (low CD8^+^ T-cell density in the IT region and high CD8^+^ T-cell density in the IM and PS regions), and immune-desert (low CD8^+^ T-cell density in the IT, IM, and PS regions). The median number of CD8^+^ T cells per field in the IT, IM, and PS regions in PGC and MGC (41.2 cells/field) was used as a cut-off to classify low and high CD8^+^ T-cell density.

The tumors were also categorized into four types according to the T-cell density (CD4^+^ and CD8^+^ cells) and PD-L1 expression in the IT and IM regions. These categories were used to predict patients who would respond to anti-PD-1 or anti-PD-L1^12^: type I, adaptive immune resistance (high TIL density and PD-L1-positive); type II, immunological ignorance (low TIL density and PD-L1-negative); type III, intrinsic induction (low TIL density and PD-L1-positive); and type IV, immune tolerance (high TIL density and PD-L1-negative). The median numbers of CD4^+^ and CD8^+^ cells per field in the IT and IM regions in PGC and MGC (111.4 cells/field) were used as the cut-offs for classification into low and high T-cell density. PD-L1 expression was determined using the CPS, which was the number of PD-L1-stained cells divided by the total number of viable tumor cells in the IT and IM regions, multiplied by 100. Tumors were considered PD-L1-positive if the CPS ≥ 1.

### RNA extraction and RNA sequencing analysis

RNA sequencing was performed on seven paired PGC and MGC FFPE tissues. Total RNA was isolated using Trizol reagent (Invitrogen, Carlsbad, CA) and the quality assessed using an Agilent 2,100 bioanalyzer with the RNA 6,000 Nano Chip (Agilent Technologies, Amstelveen, the Netherlands). RNA was quantified using an ND-2000 Spectrophotometer (Thermo Fisher Scientific, Waltham, MA). Extracted RNA samples were processed using the QuantSeq 3′mRNA-Seq Library Prep Kit (Lexogen, Vienna, Austria) according to the manufacturer’s instructions and sequenced on an Illumina NextSeq 500 (Illumina, San Diego, CA). Differentially expressed genes were defined by *P* < 0.05 and an absolute fold change > 2. Gene sets related to T-cell activation (immune checkpoints, immune checkpoint ligands, co-stimulatory molecules, co-stimulatory ligands, and chemokines) were defined (Supplementary Table 3) and GSVA performed. GSEA was performed to assess the enrichment of an 18-gene T-cell inflamed GEP (Supplementary Table 3) that predicted the response to anti-PD-1-directed therapy^13,14^. A normalized enrichment score (NSE) was specified for the GSEA. The Gene Expression Omnibus accession number for the RNA sequencing data is GSE147043.

### Statistical analysis

The cell density results are expressed as mean ± standard deviation. Non-parametric Wilcoxon signed rank tests were used to compare cell density between PGC and MGC. OS was defined as the duration between metastatic disease diagnosis and death from any cause or the last follow-up visit. Survival analysis was performed using the Kaplan–Meier method, and statistical differences were assessed by log-rank tests. Univariable and multivariable analyses were performed using the Cox proportional hazard regression model and are presented as HRs and 95% CIs for each variable. Statistical analyses were performed using IBM SPSS Statistics version 21 (IBM Corporation, Armonk, NY, USA). All statistical analyses were two-sided tests with significance defined as *P* < 0.05.

## Supplementary information

Supplementary Information.

## Data Availability

The data that support the findings of this study are available from the corresponding authors upon reasonable request.

## References

[1] Guideline Committee of the Korean Gastric Cancer Association (KGCA), Development Working Group & Review Panel (2019). Korean practice guideline for gastric cancer 2018: an evidence-based, multi-disciplinary approach. J. Gastric Cancer.

[2] Wu YL (2019). Pan-Asian adapted Clinical Practice Guidelines for the management of patients with metastatic non-small-cell lung cancer: a CSCO-ESMO initiative endorsed by JSMO, KSMO, MOS, SSO and TOS. Ann. Oncol..

[3] Postow MA, Callahan MK, Wolchok JD (2015). Immune checkpoint blockade in cancer therapy. J. Clin. Oncol..

[4] Kang YK (2017). Nivolumab in patients with advanced gastric or gastro-oesophageal junction cancer refractory to, or intolerant of, at least two previous chemotherapy regimens (ONO-4538-12, ATTRACTION-2): a randomised, double-blind, placebo-controlled, phase 3 trial. Lancet.

[5] Muro K (2016). Pembrolizumab for patients with PD-L1-positive advanced gastric cancer (KEYNOTE-012): a multicentre, open-label, phase 1b trial. Lancet Oncol..

[6] Fuchs CS (2018). Safety and efficacy of pembrolizumab monotherapy in patients with previously treated advanced gastric and gastroesophageal junction cancer: phase 2 clinical KEYNOTE-059 trial. JAMA Oncol..

[7] Kim ST (2018). Comprehensive molecular characterization of clinical responses to PD-1 inhibition in metastatic gastric cancer. Nat. Med..

[8] Zhang D (2019). Scoring system for tumor-infiltrating lymphocytes and its prognostic value for gastric cancer. Front. Immunol..

[9] Fridman WH, Zitvogel L, Sautès-Fridman C, Kroemer G (2017). The immune contexture in cancer prognosis and treatment. Nat. Rev. Clin. Oncol..

[10] Sharma P, Allison JP (2015). The future of immune checkpoint therapy. Science.

[11] Hegde PS, Karanikas V, Evers S (2016). The where, the when, and the how of immune monitoring for cancer immunotherapies in the era of checkpoint inhibition. Clin. Cancer Res..

[12] Teng MW, Ngiow SF, Ribas A, Smyth MJ (2015). Classifying Cancers Based on T-cell Infiltration and PD-L1. Cancer Res..

[13] Ayers M (2017). IFN-γ-related mRNA profile predicts clinical response to PD-1 blockade. J. Clin. Investig..

[14] Cristescu R (2018). Pan-tumor genomic biomarkers for PD-1 checkpoint blockade-based immunotherapy. Science.

[15] Ogiy R (2016). Comparison of tumor-infiltrating lymphocytes between primary and metastatic tumors in breast cancer patients. Cancer Sci..

[16] Baine MK (2015). Characterization of tumor infiltrating lymphocytes in paired primary and metastatic renal cell carcinoma specimens. Oncotarget.

[17] Zhou J (2018). Programmed death ligand 1 expression and CD8_+_ tumor-infiltrating lymphocyte density differences between paired primary and brain metastatic lesions in non-small cell lung cancer. Biochem. Biophys. Res. Commun..

[18] Haffner MC (2018). Comprehensive evaluation of programmed death-ligand 1 expression in primary and metastatic prostate cancer. Am. J. Pathol..

[19] Tawfik O, Kimler BF, Karnik T, Shehata P (2018). Clinicopathological correlation of PD-L1 expression in primary and metastatic breast cancer and infiltrating immune cells. Hum. Pathol..

[20] Eckstein M, Sikic D, Strissel PL, Erlmeier F, BRIDGE Consortium Germany (2018). Evolution of PD-1 and PD-L1 gene and protein expression in primary tumors and corresponding liver metastases of metastatic bladder cancer. Eur. Urol..

[21] Zou W, Wolchok JD, Chen L (2016). PD-L1 (B7–H1) and PD-1 pathway blockade for cancer therapy: Mechanisms, response biomarkers, and combinations. Sci. Transl. Med..

[22] Cancer Genome Atlas Research Network (2014). Comprehensive molecular characterization of gastric adenocarcinoma. Nature.

[23] Droeser RA (2013). Clinical impact of programmed cell death ligand 1 expression in colorectal cancer. Eur. J. Cancer..

[24] Xing X (2017). Analysis of PD1, PDL1, PDL2 expression and T cells infiltration in 1014 gastric cancer patients. Oncoimmunology.

[25] Wang Y (2018). PD-L1 Expression and CD8_+_ T cell infiltration predict a favorable prognosis in advanced gastric cancer. J. Immunol. Res..

[26] Marabelle A (2020). Efficacy of pembrolizumab in patients with noncolorectal high microsatellite instability/mismatch repair-deficient cancer: results from the phase II KEYNOTE-158 study. J. Clin. Oncol..

[27] Marcus L, Lemery SJ, Keegan P, Pazdur R (2019). FDA approval summary: pembrolizumab for the treatment of microsatellite instability-high solid tumors. Clin. Cancer Res..

[28] Janjigian YY (2018). CheckMate-032 study: efficacy and safety of nivolumab and nivolumab plus ipilimumab in patients with metastatic esophagogastric cancer. J. Clin. Oncol..

[29] Boku N (2019). Safety and efficacy of nivolumab in combination with S-1/capecitabine plus oxaliplatin in patients with previously untreated, unresectable, advanced, or recurrent gastric/gastroesophageal junction cancer: interim results of a randomized, phase II trial (ATTRACTION-4). Ann. Oncol..

[30] Bang YJ (2019). Pembrolizumab alone or in combination with chemotherapy as first-line therapy for patients with advanced gastric or gastroesophageal junction adenocarcinoma: results from the phase II nonrandomized KEYNOTE-059 study. Gastric Cancer.

[31] Herbst RS (2019). Ramucirumab plus pembrolizumab in patients with previously treated advanced non-small-cell lung cancer, gastro-oesophageal cancer, or urothelial carcinomas (JVDF): a multicohort, non-randomised, open-label, phase 1a/b trial. Lancet Oncol..

[32] Kwak Y (2016). Immunoscore encompassing CD3+ and CD8+ T cell densities in distant metastasis is a robust prognostic marker for advanced colorectal cancer. Oncotarget.

[33] Huang M (2020). The influence of immune heterogeneity on the effectiveness of immune checkpoint inhibitors in multifocal hepatocellular carcinomas. Clin. Cancer Res..

