# Supplementary material for: Distinct tumor immune microenvironments in primary and metastatic lesions in gastric cancer patients
Source: Sci Rep. 2020 Aug 31;10:14293. doi: 10.1038/s41598-020-71340-z (PMC7459099; doi:10.1038/s41598-020-71340-z)
Supplement: Supplementary file 1 — Supplementary Information. [file 41598_2020_71340_MOESM1_ESM.pdf]

Supplementary material

Distinct tumor immune microenvironments in primary and metastatic lesions in gastric cancer patients

Seung-Myoung Son[1,2], Chang Gok Woo[1], Dae Hoon Kim[3], Hyo Yung Yun[3,4], Hongsik Kim[5], Hee Kyung Kim[5], Yaewon Yang[5], Jihyun Kwon[5,6], Minsuk Kwon[7], Tae-Yong Kim[8], Hyung-Don Kim[9], June-Young Koh[10], Su-Hyung Park[10], Eui-Cheol Shin[10*], Hye Sook Han[5,6*]

[1]Department of Pathology, Chungbuk National University Hospital, Cheongju, Republic of Korea

[2]Department of Pathology, Chungbuk National University College of Medicine, Cheongju, Republic of Korea

[3]Department of Surgery, Chungbuk National University Hospital, Cheongju, Republic of Korea

[4]Department of Surgery, Chungbuk National University College of Medicine, Cheongju, Republic of Korea

[5]Department of Internal Medicine, Chungbuk National University Hospital, Cheongju, Republic of Korea

[6]Department of Internal Medicine, Chungbuk National University College of Medicine, Cheongju, Republic of Korea

[7]Division of Hematology-Oncology, Department of Internal Medicine, Samsung Medical Center, Sungkyunkwan University School of Medicine, Seoul, Republic of Korea

[8]Department of Internal Medicine, Seoul National University Hospital, Seoul National University College of Medicine, Seoul, Republic of Korea

[9]Department of Internal Medicine, Asan Medical Center, University of Ulsan College of Medicine, Seoul, Republic of Korea

[10]Graduate School of Medical Science and Engineering, Korea Advanced Institute of Science and Technology, Daejeon, Republic of Korea

Contents:

Supplementary Figures 1 – 3

Supplementary Tables 1 – 3

Supplementary Figure 1

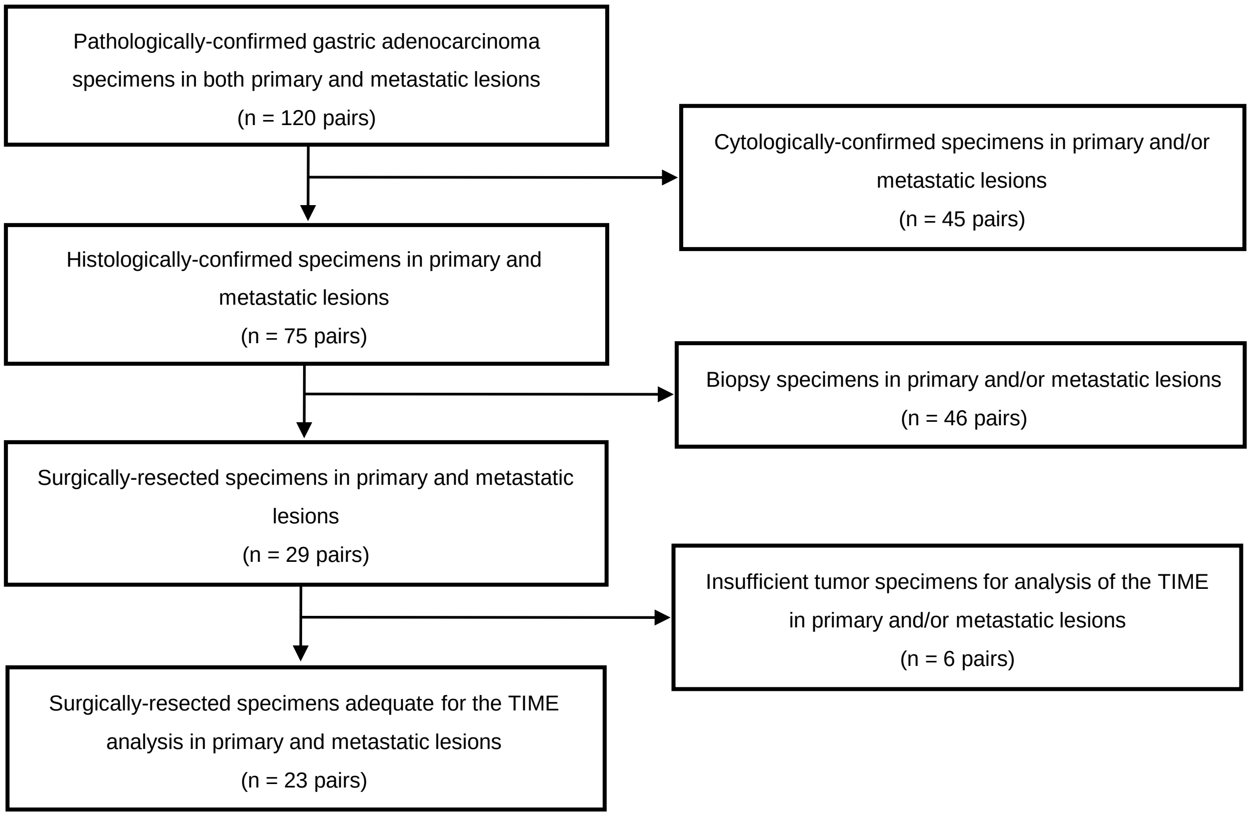

Supplementary Figure 1. Flow diagram of study inclusion. TIME, tumor immune microenvironment.

Supplementary Figure 2

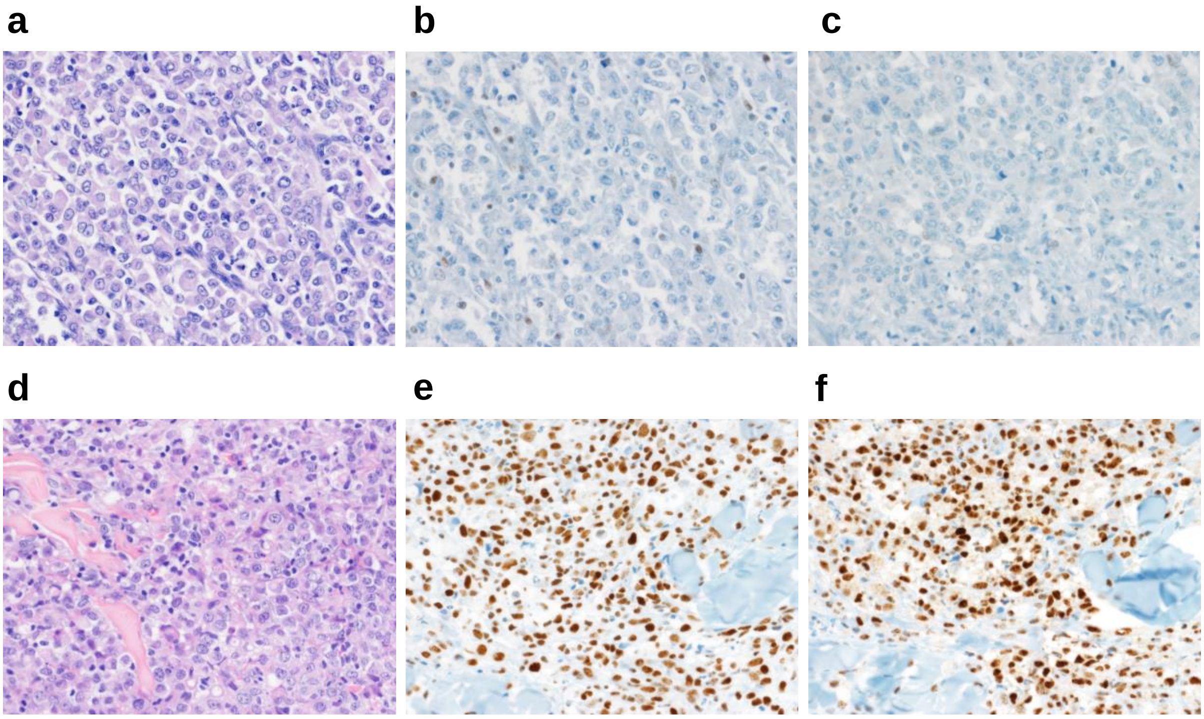

Supplementary Figure 2. Pathological features of resected primary gastric cancer and excisional biopsy of the skin metastasis. (a) The primary gastric cancer showing poorly differentiated adenocarcinoma (H&E, ×400) and loss of (b) MLH1 and (c) PMS2 protein expression (×400). (d) Excisional biopsy of the skin metastatic tumor showing poorly differentiated adenocarcinoma (H&E, ×400), but intact (e) MLH1 and (f) PMS2 protein (×400).

Supplementary Figure 3

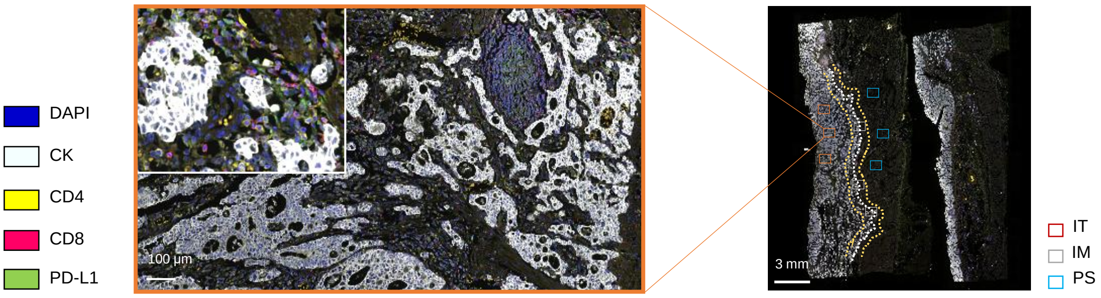

Supplementary Figure 3. Identification and characterization of T-cell infiltration and PD-L1 expression by OPAL multiplex IHC. Regions of interest consisted of 3 regions for each tumor specimen and 3 non-overlapping fields per region (one microscopic field, 925 × 693 μm). A 500 μm width on each side of the tumor margin (white dotted line) is designated as the invasive margin (IM, yellow dotted line). The intratumoral (IT) region (red) is designated as the tumor parenchyma inside the IM, and a peritumoral stroma (PS) (blue) was designated within the peritumoral region outside the IM. The total cell number in each region was calculated by the sum of the immunoreactive cells in 3 selected fields per region. OPAL multiplex IHC staining for T-cell infiltration and PD-L1 expression consisted of CK (white), CD4 (yellow), CD8 (red), PD-L1 (green) and nuclear DNA (DAPI, blue). PD-L1, programmed death-ligand 1; IHC, immunohistochemistry; CK, cytokeratin.

Supplementary Table 1. Comparison of T-cell density and PD-L1 expression between PGC and MGC

	Density (cells per field)					
	IT and IM regions			IT, IM, and PS regions		
Total (n=23)	PGC	MGC	P-value	PGC	MGC	P-value
T-cell density						
$CD4^+$	62.2 ± 60.2	44.5 ± 71.0	0.200	47.5 ± 42.1	39.4 ± 49.0	0.615
$CD8^+$	79.6 ± 73.1	35.5 ± 52.8	0.046	61.0 ± 51.2	28.5 ± 37.5	0.039
$CD4^+$ and $CD8^+$	141.8 ± 120.4	80.0 ± 107.0	0.095	108.5 ± 82.9	67.9 ± 73.8	0.131
PD-L1 expression						
$PD\text{-}L1^+$	77.5 ± 154.0	6.2 ± 18.6	0.010	52.7 ± 103.0	4.3 ± 12.8	0.001
$PD\text{-}L1^+$ and CK^+	58.8 ± 114.7	4.5 ± 13.3	0.001	40.1 ± 76.8	3.2 ± 9.2	0.001
Simultaneous resection (n=7)	PGC	MGC	P-value	PGC	MGC	P-value
T-cell density						
$CD4^+$	49.2 ± 62.3	26.6 ± 22.0	0.398	37.0 ± 41.3	27.6 ± 20.0	0.735
$CD8^+$	91.4 ± 92.8	61.2 ± 71.5	0.612	70.2 ± 64.6	51.1 ± 52.3	0.612
$CD4^+$ and $CD8^+$	140.6 ± 151.1	87.8 ± 58.9	0.612	107.2 ± 102.9	78.6 ± 66.6	0.612
PD-L1 expression						
$PD\text{-}L1^+$	36.6 ± 42.0	2.3 ± 4.0	0.043	24.8 ± 28.4	1.5 ± 2.7	0.043
$PD\text{-}L1^+$ and CK^+	21.0 ± 20.0	2.0 ± 3.5	0.043	14.3 ± 13.5	1.3 ± 2.4	0.043
Staged resection (n=16)	PGC	MGC	P-value	PGC	MGC	P-value
T-cell density						
$CD4^+$	68.3 ± 60.5	52.9 ± 84.3	0.363	52.4 ± 43.0	44.8 ± 57.6	0.733
$CD8^+$	74.0 ± 65.0	23.5 ± 38.9	0.015	56.8 ± 43.9	18.0 ± 26.0	0.008
$CD4^+$ and $CD8^+$	142.3 ± 109.3	76.4 ± 118.1	0.078	109.2 ± 76.0	62.8 ± 78.7	0.100

PD-L1 expression						
PD-L1^{+}	96.6 ± 183.3	8.0 ± 22.4	0.011	65.7 ± 122.4	5.6 ± 15.5	0.011
PD-L1^{+} and CK^{+}	76.5 ± 136.1	5.7 ± 16.0	0.011	52.1 ± 91.0	4.0 ± 11.1	0.011
No systemic therapy before MGC resection (n=18)	PGC	MGC	P-value	PGC	MGC	P-value
T-cell density						
CD4^{+}	57.5 ± 61.3	40.0 ± 72.6	0.286	43.7 ± 42.2	33.7 ± 49.3	0.586
CD8^{+}	83.5 ± 80.4	41.5 ± 56.8	0.133	62.6 ± 55.1	33.0 ± 41.3	0.133
CD4^{+} and CD8^{+}	141.0 ± 131.1	81.5 ± 115.6	0.199	106.3 ± 89.2	66.7 ± 80.0	0.215
PD-L1 expression						
PD-L1^{+}	87.0 ± 1 67.9	7.3 ± 20.5	0.003	59.2 ± 112.3	5.1 ± 14.1	0.003
PD-L1^{+} and CK^{+}	64.6 ± 124.6	5.3 ± 14.6	0.003	44.0 ± 83.5	3.7 ± 10.1	0.003
Systemic therapy before MGC resection (n=5)	PGC	MGC	P-value	PGC	MGC	P-value
T-cell density						
CD4^{+}	83.7 ± 57.7	65.1 ± 68.5	0.465	64.6 ± 43.1	64.8 ± 44.3	1.000
CD8^{+}	61.9 ± 17.0	8.4 ± 8.0	0.068	53.9 ± 18.7	8.2 ± 3.7	0.068
CD4^{+} and CD8^{+}	145.6 ± 63.2	73.5 ± 65.3	0.273	118.5 ± 53.8	72.9 ± 43.2	0.273
PD-L1 expression						
PD-L1^{+}	34.5 ± 57.1	1.2 ± 2.2	0.273	23.6 ± 37.8	0.8 ± 1.5	0.273
PD-L1^{+} and CK^{+}	32.8 ± 54.6	1.1 ± 2.0	0.273	22.5 ± 36.2	0.8 ± 1.4	0.273

Data are given as mean ± standard deviation. IT, intratumoral; IM, invasive margin; PS, peritumoral stroma; PGC, primary gastric cancer; MGC, metastatic gastric cancer; PD-L1, programmed death-ligand 1; CK, cytokeratin

Supplementary Table 2. Comparison of tumor immune microenvironment types between PGC and MGC

	No. of patients (%)		
	PGC	MGC	Total
Types based on $CD8^{+}$ T-cell density and distribution			
Inflamed	8 (34.8)	6 (26.1)	14 (30.4)
Immune excluded	7 (30.4)	2 (8.7)	9 (19.6)
Immune desert	8 (34.8)	15 (65.2)	23 (50.0)
Types based on the TIL density and PD-L1 expression			
Type I, Adaptive immune resistance	8 (34.8)	0	8 (17.4)
Type II, Immunological ignorance	7 (30.4)	17 (73.9)	24 (52.2)
Type III, Intrinsic induction	4 (17.4)	1 (4.3)	5 (10.9)
Type IV, Immune tolerance	4 (17.4)	5 (21.7)	9 (19.6)

PGC, primary gastric cancer; MGC, metastatic gastric cancer; TIL, tumor-infiltrating lymphocytes

Supplementary Table 3. Genes used in Gene Set Analysis

Gene set	Gene	Gene set	Gene
Immune checkpoints	*PDCD1*	Immune checkpoint ligands	*CD274*
	CTLA4		*PDCD1LG2*
	HAVCR2		*CD80*
	TIGIT		*CD86*
	LAG3		*LGALS9*
	BTLA		*PVR*
	ADORA2A-AS1		*CD276*
	KIR2DL1		*VTCN1*
	KIR2DL3		*TNFRSF14*
	KIR2DL4		*HLA-A*
	KIR2DS4		*HLA-B*
	KIR3DL2		*HLA-C*
			HLA-DPA1
			HLA-DPB1
			HLA-DQA1
			HLA-DQB1
			HLA-DRA
			HLA-DRB1
Co-stimulatory molecules	*CD28*	Co-stimulatory ligands	*CD80*
	ICOS		*ICOSLG*
	CD40LG		*CD40*
	TNFRSF9		*TNFSF9*
	CD27		*CD70*
	TNFRSF4		*TNFSF4*
	TNFRSF18		*CD47*
	SIRPA		
Chemokines	*XCL1*	18-gene T-cell inflamed GEP	*TIGIT*
	XCL2		*CD27*
	CX3CL1		*CD8A*
	CCL1		*PDCD1LG2*
	CCL2		*LAG3*
	CCL3		*CD274*
	CCL3L1		*CXCR6*
	CCL3L3		*CMKLR1*
	CCL4		*NKG7*
	CCL4L1		*CCL5*

	CCL4L2	*PSMB10*
	CCL5	*IDO1*
	CCL7	*CXCL9*
	CCL8	*HLA-DQA1*
	CCL15	*CD276*
	CCL11	*STAT1*
	CCL14	*HLA-DRB1*
	CCL16	*HLA-E*
	CCL17	
	CCL18	
	CCL19	
	CCL20	
	CCL21	
	CCL22	
	CCL27	
	CCL28	
	CXCL1	
	CXCL2	
	CXCL3	
	PF4V1	
	CXCL5	
	CXCL6	
	CXCL10	
	CXCL11	
	CXCL12	
	CXCL13	
	CXCL14	
	CXCL16	
	CXCL17	
